# Bromodomain-Containing Protein 4: A Dynamic Regulator of Breast Cancer Metastasis through Modulation of the Extracellular Matrix

**DOI:** 10.1155/2012/670632

**Published:** 2011-10-26

**Authors:** Jude Alsarraj, Kent W. Hunter

**Affiliations:** Laboratory of Cancer Biology and Genetics, National Cancer Institute, National Institutes of Health, Bethesda, MD 20892, USA

## Abstract

Metastasis is an extremely complex process that accounts for most cancer-related deaths. Malignant primary tumors can be removed surgically, but the cells that migrate, invade, and proliferate at distant organs are often the cells that prove most difficult to target therapeutically. There is growing evidence that host factors outside of the primary tumors are of major importance in the development of metastasis. Recently, we have shown that the bromodomain-containing protein 4 or bromodomain 4 (*Brd4*) functions as an inherited susceptibility gene for breast cancer progression and metastasis. In this paper, we will discuss that host genetic background on which a tumor arises can significantly alter the biology of the subsequent metastatic disease, and we will focus on the role of *Brd4* in regulating metastasis susceptibility.

## 1. Introduction

Breast cancer is the most common cancer diagnosed in women worldwide. In the United States the estimates for 2010 were 209,060 new cases of invasive breast cancer and 40,230 deaths [1]. The main cause of breast cancer-related deaths is metastatic disease. The overall 5-year relative survival of patients with metastatic breast cancer is 23%, while the relative survival of breast cancer patients with nonmetastatic tumors is 98% [2]. Patients who have no evidence of tumor dissemination at the time of diagnosis are still at risk of metastatic disease. Approximately one-third of women who are sentinel lymph node negative at the time of surgical resection of the breast primary tumor will eventually develop clinically detectable secondary tumors [3]. Therefore, understanding the mechanisms governing tumor dissemination and developing new strategies to control or effectively treat patients with or at risk of metastatic disease would significantly improve the overall outcome of the disease. 

**Figure 1:**
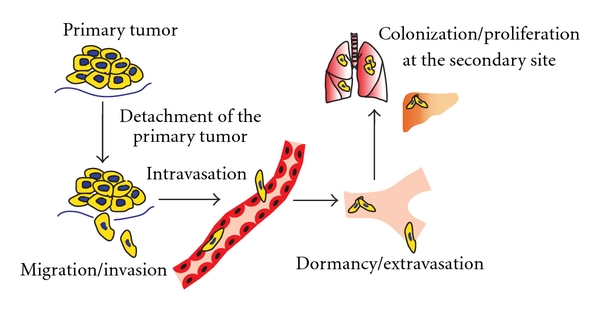
The steps of the metastatic cascade.

Metastasis is a multistep complex process that involves the detachment of tumor cells from the primary tumor, migration and invasion through the surrounding tissues and basement membranes, intravasation and survival in the small blood vessels or lymphatic channels, and colonization in a distant target organ. These steps are usually followed by extravasation into the surrounding tissue, survival in the foreign microenvironment, proliferation, and induction of angiogenesis (Figure 1). It has become apparent that the vast majority of tumor cells within the primary tumor and also the disseminated tumor cells will not form distant metastases, either because they die or remain dormant [4]. The dormancy phenomenon probably explains what is seen in the clinic in which some cancer patients remain free of clinical evidence of metastatic disease for years or even decades after primary tumor resection, and after this prolonged period of time these patients show signs of tumor relapse. The development of the primary tumor microenvironment is also an important determinant of tumor dissemination; this tumor microenvironment may influence the release of cancer cells into the blood and the lymphatic systems and subsequently promote continued survival and proliferation at the secondary site. It has been well known that the interaction between tumor cells and their microenvironment is important for establishing metastatic colonies and for defining the balance between dormancy and malignant growth [5, 6]. Furthermore, the microenvironment of metastatic tumor cells has recently been thought to play a major role in tumor progression. Although tumor cells may be continually released from the primary site, relatively few of them are able to efficiently form macrometastasis. What are the factors controlling this important step?

In this paper we will discuss a more global view of host-tumor interactions in which the metastatic potential of tumors is an inherent component of cells forming the primary tumor mass at an early time in tumor progression. We will also discuss the association of two genes, *Brd4* and *Sipa1 *(signal-induced proliferation-associated 1), with mammary tumor progression in both the mouse and the human. Here, we will focus on the role that the metastasis susceptibility gene *Brd4* plays in the regulation of extracellular matrix (ECM) gene expression and subsequently metastatic progression. 

## 2. Genetic Background Plays an Important Role in Metastasis

Studies from our laboratory have demonstrated that the inherited polymorphism, or the genetic background on which a tumor arises, plays an important role in determining the probability that the given tumor will progress to metastatic disease. These findings are based on a series of genetic mapping studies using the highly metastatic polyoma middle-T (PyMT) transgenic mammary mouse model. This mouse expresses the mouse polyoma virus middle-T antigen in the mammary epithelium of FVB/N inbred mice [7] from an early age [8], which results in the development of highly aggressive mammary tumors that metastasize to the lung with high frequency [7]. Specifically, when the male PyMT transgenic mouse was bred to different inbred strains, the F1 progeny showed significant variation in tumor characteristics, such as tumor latency, growth kinetics, and metastatic capacity [9]. It is important to point out that these tumors were all induced by the same oncogenic event, the activation of the PyMT transgene. Subsequent analysis identified several interacting quantitative trait loci (QTL), some of which were found to map to homologous regions associated with loss of heterozygosity in human breast cancer [8, 10, 11]. Together, these findings suggest that inherited germline polymorphisms may contribute to the age of onset in human breast cancer and also the ability of tumor cells to metastasize. Further investigation of these earlier observations identified the first known polymorphic metastasis susceptibility gene, the Rap-GTPase activating protein (GAP) SIPA1 [12]. Experimental manipulation of cellular *Sipa1* mRNA levels in a highly metastatic mouse mammary tumor cell line showed that subtle differences in *Sipa1* levels significantly affected the ability of the cells to colonize to the lungs, while not impacting primary tumor kinetics [12]. Studies of human breast cancer have suggested that *SIPA1* germline polymorphisms are associated with aggressive disease behavior and with indicators of poor prognosis [13, 14], suggesting that *Sipa1* may play an important role in establishing metastatic susceptibility in humans as well as in mice. 

## 3. Inherited Polymorphisms and ECM Gene Expression Profile

We have shown so far that hereditary polymorphisms modulate metastatic potential. To further study whether genetic polymorphisms could be an important factor in the induction of prognostic signature profiles, previously published metastasis-predictive gene expression signatures were examined both in the mouse and in humans. ECM genes were found to be common components of the metastasis-predictive gene signature in both human breast tumors [15–17] and in PyMT-induced mouse mammary tumors [18, 19], suggesting an important association of these genes with breast cancer progression. Briefly, the ECM components constitute a structure that is not only essential for the maintenance of tissue integrity but is also important for regulating cell migration. Historically, tumor interaction with the basement membrane was defined as the critical event in tumor invasion that signals the initiation of the metastatic cascade. Many steps in metastasis formation require specific interactions with the ECM [20]. The nature and degree of this interaction will change from step to step during the metastatic process. However, the type of specific interactions between tumor cells and the ECM might be influenced by the type of tumor cells and the type of matrix in which they reside. For example, tumor cells may respond differently to various extracellular matrices and stromal cells that are encountered during metastasis formation, and this might result in the emphasis of some steps over others at particular points in the metastatic cascade (reviewed in [21]). 

A study by Bergamaschi and colleagues has portrayed the tumor-stroma composition of invasive breast carcinomas by characterizing the ECM components [22]. Differential expression of ECM-related genes identified four distinct groups. The ECM classification was recapitulated in a set of early-stage primary breast carcinomas [22, 23]. Survival analysis on the early-stage breast carcinoma dataset showed significant differences in clinical outcome among the various ECM subclasses [22]. Several studies that explored gene expression differences of primary breast and metastatic lymph node tumors have shown that genes involved in changes in extracellular matrix stability are critical for the early stages of the metastatic process [24–27]. Furthermore, ECM gene dysregulation has been shown to be a very prominent feature of metastatic progression and may well explain why highly metastatic mouse mammary tumor cell lines are typically more adhesive, invasive, and migratory than the less metastatic lines [28]. To determine whether the ECM dysregulation is under germline control, the AKXD recombinant inbred mice (RI) [29] were used to define ECM expression quantitative trait loci (eQTL). An eQTL is a genetically defined genomic locus associated with variation of gene expression, in this case ECM gene expression [30]. We chose the AKXD RI mice because they are considered a useful tool for the study of germline-encoded metastatic propensity since they are derived from a highly metastatic strain, AKR/J, and a weakly metastatic strain, DBA/2J [9]. We found that the most significant eQTL in these mice is located on proximal mouse chromosome 17. This eQTL colocalizes to the peak region of linkage of a metastasis susceptibility QTL [31]. Both of these eQTL and metastasis loci colocalize and reside in a genomic region that contains the gene* Brd4*, suggesting that *Brd4* modulates ECM gene expression. 

## 4. *Brd4* Is a Potential Metastasis Susceptibility Gene

*BRD4* is the mammalian member of the BET (bromodomain and extra-terminal) family [32, 33], whose members carry two tandem bromodomains [34, 35]. BRD4 has been shown to regulate cell growth by acting at different stages of the cell cycle and also to interact with acetylated chromatin through its two bromodomains [32, 33]. Given the apparent modulation of ECM gene expression, we further investigated the possibility that *Brd4* might be a metastasis susceptibility gene. Indeed, we found that ectopic expression of *Brd4* in a highly metastatic mouse mammary tumor cell line reduces both primary tumor growth and metastatic capacity in our mouse model [36]. *In vitro* analyses showed that *Brd4 *ectopic expression reduces both cell invasion and cell migration and also reduces cellular growth in three-dimensional cultures [36]. These data are consistent with our previous findings that *Brd4* modulates ECM gene expression. Microarray gene expression analysis of the cell lines ectopically expressing *Brd4*, further confirmed that *Brd4* is a regulator of at least some of the ECM gene family members [36]. Some of the ECM genes that were altered by ectopic expression of *Brd4* are the collagen genes *Col1a1*, *Col5a3*, *Col6a2*, the fibrillin gene *Fbn1 *and *Serping1*, indicating that *Brd4* is a causative factor in the transcriptional regulation of these genes [36].

### 4.1. *Brd4* and *Sipa1* Interaction and Metastatic Progression

BRD4 has been previously found to interact *in vitro* and *in vivo* with the metastasis modifier SIPA1 [37]. This interaction modulates the enzymatic activity of SIPA1 by increasing its RAP-GAP activity. The N-terminus bromodomain II of BRD4 was shown to be the domain where BRD4 and SIPA1 interact [37]. Deletion of bromodomain II resulted in further suppression of primary tumor growth and lung metastasis mediated by* Brd4* and also induced a conversion to a more epithelial state [38]. These results are consistent with our previous findings that SIPA1 is associated with greater malignancy [12]. It is important to mention here that BRD4 and SIPA1 were shown to regulate each other's subcellular localization, with BRD4 being redirected from the nucleus to the cytoplasm [37]. It is possible that the interaction between these two proteins contributes to tumor progression, and also the activity of *Brd4 *might be modulated by compartmentalization; however, the mechanism by which this occurs has yet to be explored. One possibility could be that there is a balance between BRD4 and SIPA1 within the cell. Under normal conditions BRD4 and SIPA1 interact in the nucleus while the cytoplasmic SIPA1 does not take part in this interaction. Upon *Brd4* overexpression, SIPA1 accumulates in the perinuclear region and in some cases in the nucleus near the nuclear membrane [37]. However, when *Sipa1* is overexpressed, a large fraction of BRD4 gets moved to the cytoplasm leading to a more malignant phenotype. Our results suggest that the loss of the ability of SIPA1 to relocalize or sequester the bromodomain II mutant to the cytoplasm would increase the nuclear concentration of BRD4, leading to a more differentiated state and a less malignant phenotype. At this point it is not known whether the BRD4-SIPA1 interaction influences the small GTPase RAP1 levels within the tumor cell. RAP1 activity has been shown to play an important role in tumor formation and progression to malignancy [39, 40]. Further investigations of the BRD4-SIPA1 relationship and the influence that it could have on RAP1 levels might reveal a novel mechanism associated with malignant progression.

### 4.2. *Brd4* and Regulation of Epithelial-to-Mesenchymal Transition (EMT)

BRD4 is known to be a transcriptional regulator. As mentioned earlier BRD4 contains two bromodomains that bind acetylated histones [32]. A recent report has shown that the extraterminal (ET) domain of BRD4 is an important transcriptional regulatory domain [41]. The C-terminal domain contains a single defined domain that binds the transcriptional elongation factor P-TEFb [42]. BRD4 also contains regions of high serine, proline, and glutamine content of unknown function. Indeed, microarray gene expression analysis of the cell lines that ectopically express *Brd4* has revealed that *Brd4* modulates the expression of genes involved in processes such as cellular proliferation, cell cycle progression, and chromatin remodeling [36]. Other processes that are critical for metastasis, such as cytoskeletal remodeling, cell adhesion, and as mentioned earlier ECM expression regulation, were also regulated by *Brd4 *[36]. Furthermore, microarray gene expression analysis of cell lines that express a C-terminal deletion of *Brd4* show modulation of other classes of genes involved in EMT and stem cell conversion processes [38].

 EMT is a multigstep process in which the cells acquire molecular changes that lead to a loss of cell-cell junctions, dysfunctional cell-cell adhesion, and rearrangement of the cytoskeleton, leading to a loss of polarity and the acquisition of a more spindle-shape morphology [43–48]. These alterations might eventually promote cancer cell progression and invasion through the basement membrane and into the surrounding tissues. Indeed, several studies have associated EMT with cancer progression and metastasis [49–52]. For example, EMT markers have been found to be present in invasive breast cancer especially in the invasion-metastasis cascade [47, 53]. Recently, a concept of the “migratory cancer stem cell” has been described [54], in which a tumor cell possesses both stemness and motility properties. It is suggested that cancer stem cells that have undergone EMT can disseminate, and those that retain stem-cell functionality can form metastatic colonies [54]. More recently the EMT process has also been linked to the ability of self-renewal [55]. Current thinking suggests that disseminated cancer cells may need to acquire self-renewal properties similar to those exhibited by the stem cells, in order to achieve formation of macroscopic metastases [55]. The role of EMT in tumor-initiating cells has also been described in human specimens. Breast cancer tumor-initiating cells and mesenchymal claudin-low-subtype cells show an association based on gene expression pattern [56]. Furthermore, higher expression of mesenchymal genes was detected in breast cancer tumors before and after treatment with letrozole, indicating that the epithelial cancer cells have undergone EMT [56]. 

Ectopic expression of the C-terminal deletion mutant of *Brd4* (ΔC) in a highly metastatic cell line induced significant morphological and physiological changes reminiscent of EMT-like and cancer stem cell-like properties [38]. Microarray gene expression analysis of these cell lines demonstrated that ectopic expression of the ΔC mutant modulated the expression of some previously described EMT markers and stem cell markers. It is important to point out here that this mutant still contains the P-TEFb-binding domain suggesting that EMT-like and stem cell-like changes appear to be mediated by this P-TEFb-binding region. The mechanism on how this might occur is currently under investigation. 

### 4.3. *Brd4* Isoforms and Metastasis Regulation

*Brd4 *has two alternatively spliced variants that differ in the coding region and have a distinct 3′ UTR. Both isoforms have the same N-terminal region containing the chromatin-binding bromodomains and the serine-rich domain; however, the C-terminal proline-rich and P-TEFb-binding domains are absent in the shorter isoform. We have found that ectopic expression of the short isoform enhances metastatic colonization [38], as opposed to that seen by ectopic expression of the longer isoform [36]. This would suggest that the *Brd4* short isoform might be a competitive inhibitor of the longer isoform and that this inhibition would increase the ability of tumors to progress to metastatic disease. This also suggests that metastatic susceptibility might be encoded by a ratio between the two isoforms. The above data also suggest that the carboxy terminal half of the full-length isoform mediates the ability of *Brd4* to suppress progression and metastasis. This was confirmed by the finding that expression of the C-terminal ΔC mutant of *Brd4* increased lung colonization [38]. This increased malignancy is consistent with the *in vitro* data that cells expressing this mutant possess EMT- and stem cell-like properties. It is not known at this point whether the ratio between the two *Brd4* isoforms influences the expression of *Sipa1* or vice versa. It would also be highly interesting to determine whether the BRD4 short isoform and SIPA1 could change each other's subcellular localization as seen with the longer isoform. The ratio between these three proteins and their cellular localization could be critical for malignant progression. 

It is important to mention here that, in rare midline carcinomas, a highly malignant form of human squamous carcinoma, the *BRD4* short isoform is frequently fused to the *NUT* (nuclear protein in testis) oncogene via an intronic translocation [57–60]. The major oncogenic effect of BRD4-NUT fusion protein appears to lie in its ability to arrest the differentiation of the so-called NUT-midline carcinoma cells [59]. This is consistent with our findings that the shorter *Brd4* isoform promotes metastatic capacity and also that the competitive inhibition of the longer *Brd4* isoform would increase the ability of tumors to progress to metastatic disease [38].

### 4.4. *Brd4* Isoforms Expression and Gene Expression Signatures

Several studies have demonstrated that primary tumors with a higher propensity to metastasize exhibit gene expression patterns that predict the likelihood of metastatic potential [15–17]. As mentioned earlier, *Brd4* is responsible, at least partially, for the presence of ECM components in the metastatic-predictive gene signatures [36], suggesting that *Brd4* itself might be a predictive of survival. We have found that the *Brd4* long isoform induces a gene expression signature that predicts good outcome in human breast cancer datasets. This suggests that *Brd4* activation is an important determinant in the overall likelihood of relapse and/or survival [36]. The* Brd4* gene expression signature was also able to stratify breast cancer patients with lymph-node-negative and estrogen-receptor-positive at presentation into high- and low-risk patients [36]. The gene expression signature induced by the *Brd4 *short isoform, however, predicted poor outcome in these human breast cancer datasets [38], confirming that the shorter isoform might be a competitive inhibitor of the longer isoform. Additionally, the *Brd4* long- and short-isoform gene expression signatures were compared to a 19-gene signature that was defined by correlating tumor growth expression, histological grade, and survival [61]. We found that the *Brd4* longer isoform signature matches low-grade G1 breast cancer tumors while the shorter isoform matches high-grade G3 tumors [36, 38]. These observations were completely consistent with our *in vivo* data. The outcome prediction and the signature convergence might be of potential importance in the clinic where it could improve the stratification of patients into different subtypes and in turn enable clinicians to tailor treatments for individual patients.

## 5. *Brd4* as a Therapeutic Target

 Selective inhibitors of the BET family members have been recently developed [62–65]. A competitive binding of the small molecule inhibitor JQ1, for example, was shown to displace the BRD4 fusion oncoprotein from chromatin, promoting squamous differentiation and specific anti-proliferative effects [63]. These effects were seen in BRD4-dependent cell lines and patient-derived xenograft models [63]. In another study, Zuber and colleagues studied acute myeloid leukemia (AML), which is an aggressive hematopoietic malignancy that is often associated with aberrant chromatin states [65]. Suppression of *Brd4* by shRNA or by JQ1 compound led to robust antileukemic effects both *in vivo* and *in vitro*. *Brd4* inhibition also led to myeloid differentiation and leukemia stem-cell depletion [65]. At this point it is not known whether the small-molecule inhibition of *Brd4* would have any effect on breast cancer and metastatic progression. However, the recent findings establish *Brd4* as a promising target for therapeutic intervention.

## 6. Conclusions

It is clear that the genetic background is an important determinant of tumor progression. The genetic background impacts not only the primary tumor but all of the tissues, which play a role in the establishment of the microenvironment in both primary and metastatic tumor cells. This would suggest that an earlier prognosis in nontumor tissues should be possible even before cancer develops. This is only possible if a sufficient fraction of metastatic risk is encoded by germline polymorphisms, rather than autonomous somatic events within the tumor. 

Our recent data suggest that the metastasis susceptibility gene *BRD4* appears to play a significant role in establishing transcriptional programs that predict breast cancer outcome via a balance between the tumor- and metastasis-suppressive long isoform and the metastasis-promoting short isoform. Given the fact that *BRD4* regulates important intermediates and processes within the metastatic cascade suggests that *BRD4*, and possibly other metastatic susceptibility genes, may be altering the risk of developing distant metastases by predisposing the tumors of high-risk patients to undergo conversion to a more dedifferentiated or primitive state. Finally, the *Brd4* gene expression signature identified could be applied as a useful predictive tool by identifying those patients with low risk of relapse at presentation. This combined with the traditional clinical variables such as lymph node-negative and ER-positive patients would facilitate the identification and the initiation of new treatment protocols that could be applied for individual patients. 

##  Glossary

**Invasion**

A process that initiates metastasis and consists of changes in tumor cell adherence to the extracellular matrix, proteolysis of the extracellular matrix and the surrounding tissues and migration through these tissues.

**Intravasation**

The entry of tumor cells into the bloodstream.

**Extravasation**

The escape of tumor cells from the circulation into the parenchyma of an organ.

**Colonization**

A process by which disseminated tumor cells grow to form clinically detectable metastatic lesions.

**Angiogenesis**

The formation of new blood vessels that are needed for the growth of the primary tumor and metastases.

**Dormancy**

A period in which the cells are in a non-dividing state.

**Polymorphism**

A variation within a gene where two or more alleles exist at a frequency of at least 1% in the general population.

**Extracellular Matrix**

The matrix that is laid down by cells in which they adhere and move.

**Expression Quantitative Trait Locus (eQTL)**

A genetically defined genomic locus associated with variation of expression of the genes that underlie the trait in question.

**Epithelial-to-Mesenchymal Transition (EMT)**

A potential mechanism in tumor progression by which some cancer cells acquire the ability to convert from polarized epithelial cells to mesenchymal motile cells facilitating metastasis at distant sites.
